# Assessment of biodiversity goods for the sustainable development of the chagra in an indigenous community of the Colombian Amazon: local values of crops

**DOI:** 10.1186/s13002-021-00453-0

**Published:** 2021-04-01

**Authors:** Giovanny Garavito, Rafael Clavijo, Pilar Luengas, Pablo Palacios, María Helena Arias

**Affiliations:** 1 Sede Bogotá, Facultad de Ciencias, Departamento de Farmacia, Grupo de Investigación FaMeTra (Farmacología de la Medicina Tradicional y Popular), Universidad Nacional de Colombia, Carrera 30 45-03, Bogotá D.C., 111311 Colombia; 2 Parque Ecológico Mundo Amazónico, km 7 Vía Tarapacá, Leticia, Colombia; 3 Sede Amazonía, Universidad Nacional de Colombia, Kilometro 2 Vía Tarapacá, Leticia, Colombia

**Keywords:** Amazon, Traditional knowledge, Chagra, Ethnobotany, Biodiversity

## Abstract

**Background:**

The chagra is the agroforestry system adapted to the characteristics of the Amazon region. Recently, there has been a reported loss of biodiversity and traditional knowledge associated with the chagras. This paper characterizes the cultivators, exploring knowledge and expressed value perception in the context of the Amazonian chagra of an indigenous community; also, this prioritizes species, under the optics of commercial opportunity.

**Methods:**

A semi-structured instrument was applied to 14 volunteers, asking about marketing preferences and use values of the species; later, a floristic inventory and prioritization workshop was developed.

**Results:**

Sixty-two percent of the participants were 50 years or older at the time of the interview. Open conversations showed that traditional knowledge is a matter of practice; and is maintained mainly by the older "grandfathers". Thirty-eight species, belonging to 28 different families, were reported, showing considerable diversity. Seventy-nine percent of the participants consider the Leticia market and sales to tourists as the main marketing scenarios.

**Conclusions:**

The Ziora-Amena community centralizes the handling of chagras in the community's older adults, who transmit their traditional knowledge to new generations through oral tradition. Indicators of preference, use, and abundance highlight the food species. The perception of the trade stakeholder encourages research and development of endemic species, with health properties or ingredients for industry, which represent an opportunity of high added value for the region.

## Introduction

Land use change will have its greatest global impact on biodiversity by 2100, and is expected to be particularly significant in the tropics [1]. The majority of the world’s population (70%) is fed and nourished by local and ecological food production systems, but these systems are severely threatened by industrial farming systems that have exacerbated multiple crises of rising food prices, poverty, climate change, and biodiversity loss [2]. Food sovereignty involves the right of indigenous peoples to healthy and culturally appropriate food produced with traditional, ecologically sustainable methods.

The chagra is the traditional agroforestry system adapted to the characteristics of Amazon environment, in which availability of biomass depends on the constant exchange of nutrients derived from the decomposition of dead material (slash and burn). It is generally an area of polyculture, transitory during the first 2 to 4 years, giving rise later to the “stubble” or animal chagras (which remain a few more years for fruit production) [3–5]; finally, natural vegetation regenerates [6]. The chagra is also a space for transmission of traditional knowledge (TK) about management, sustainable use, and conservation of biodiversity, and where education about family and social relations of the community is provided [3, 7]. Furthermore, it is a scenario of accumulation and transmission of knowledge about the handling of food, ritual, and medicinal plants through a permanent exercise of domestication, reproduction, and selection of varieties of plants.

Previous works report the loss, in recent decades, of two thirds of the species present in the chagra, a progressive loss of genetic diversity with a tendency to maintain so-called “commercial” species and a shift towards monocultures [7]. The proximity of indigenous communities to cities negatively impacts traditional farming systems and thus their food and labor sovereignty. Currently, there is a crisis in the traditional and successful farming system of the indigenous communities, due to the lack of interest and preference for foreign foods, the decrease in varieties and species present thus the inability to satisfy the food requirements of the families (generating additional pressure on the extractive methods fishing, hunting, and gathering in the forest), and the absence of policies and proposals to revitalize the chagra [6]. Additionally, this crisis threatens the exercise and transmission of TK in medicinal plants to address local etiologies, leading to increased demand for health services and the inevitable loss of valuable knowledge.

The Colombian Amazon covers 483,164 km^2^, 83% of which correspond to forests. There are 7389 plant species present in the area, of which 1575 are considered valuable, 269 of which have been introduced [8]. The sustainable use of wild flora and forests is, since 1996, a policy of the Colombian state; focused on the conservation and management of the resource, seeking to create an environment conducive to investment in environmental matters and for the development of the forestry sector; taking into account that much of the natural forest areas of the country are inhabited, it is necessary to support the satisfaction of vital needs, the conservation of their traditional values, and the exercise of the rights of its inhabitants, within the limits of the common good [9]. In light of the foregoing, there are necessary alternatives that, taking into account the social context and opportunities of the area, allow for the contextualization and maintenance of the traditional practices of the chagra, providing them with some type of stimulus for the farming families, thus reinforcing food sovereignty, the appreciation of their own knowledge and improving the quality of life.

In recent years, ethnobotanical studies have increasingly focused on finding ways to express the commercial and non-commercial values of forests to rural people [10]. First, quantitative methods were developed in an attempt to go beyond simply listing the names and uses of local plants to obtain information that indicates more about the relative values of different species. Today, the aim is to express use values in terms that explicitly reflect the importance of plants as perceived by local stakeholders, so quantifying indicators should help focus future research or interventions on the most significant species [10].

As desirable characteristics in the evaluation and selection of forest product supply, it is recommended to consider economic, social and ecological factors [11]. This work characterizes the cultivators exploring their traditional knowledge associated to the chagra, using a rapid methodology sensitive to the appreciation of individuals, to quantify the cultural relative importance that the actors give to biodiversity goods; that perception was confirmed floristically through the identification and abundance valuation of the taxa reported in the chagras. Finally, the species with the best use, importance, and abundance values were valued from the point of view of a trading user of these goods, adjusting the priority order according to economic and market differentiation criteria, with the aim of proposing priorities in the sustainable use policy that allows the recovery of the chagra.

## Methods

### Study area

**Fig. 1:**
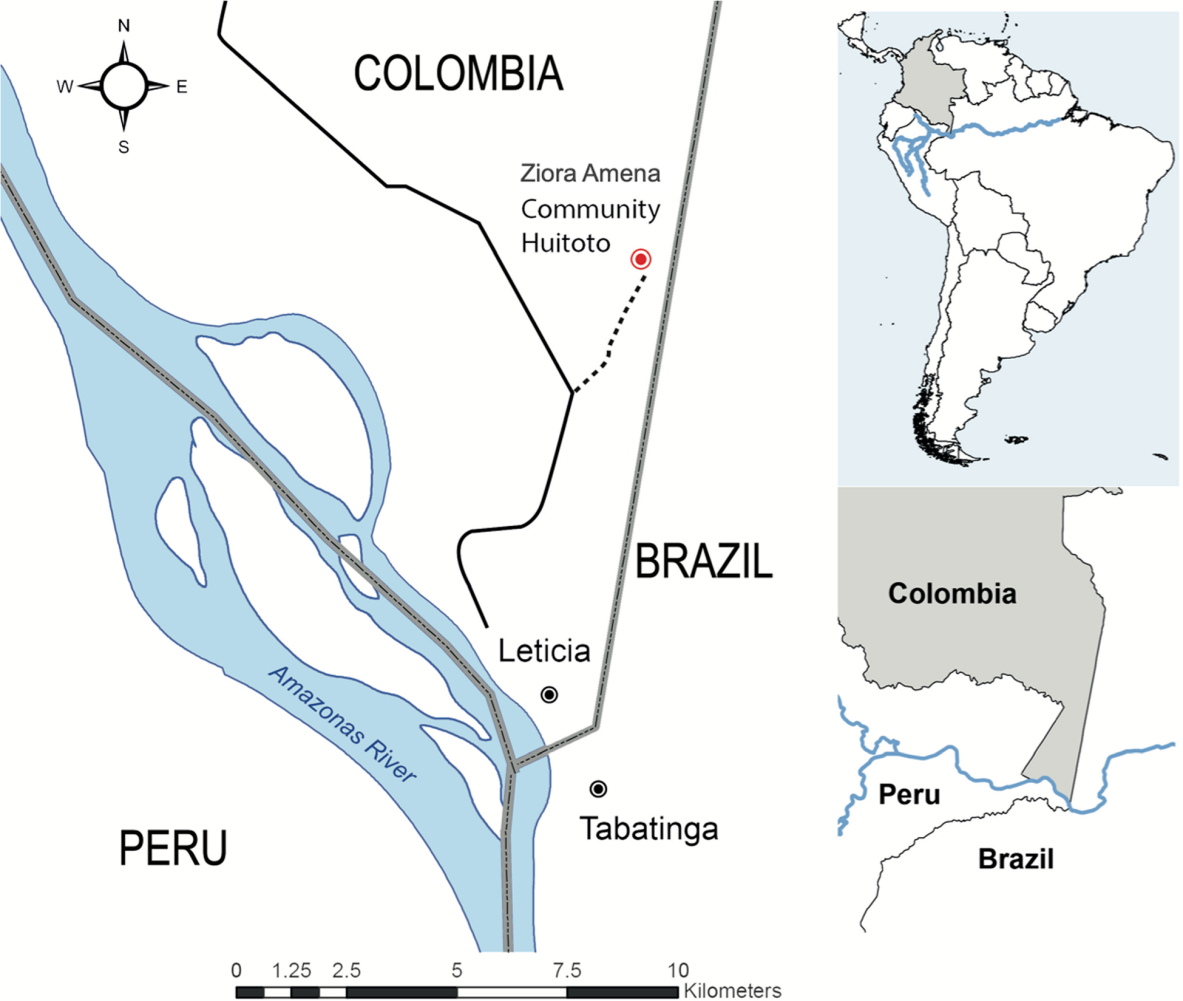
Map showing the location of the Ziora-Amena community. Adapted, with permission of the author, from Rodríguez et al. [13]

This research was carried out at the south of the state of Amazonas, in the Ziora-Amena Community, “Km 7” of the Leticia-Tarapaca road (Fig. 1), located in the lowlands of the Colombian Amazon, with a jungle climate, with an average annual rainfall of about 2846 mm/year, and an annual average temperature of 26.6 °C. Rains are constant throughout the year; there is a period of low rainfall (June to September), a higher-than-average volume of rain (November to June), and average humidity of 87.4%. The community’s territory has two tributary streams of the Yahuarcaca and Tacana streams; 60% is cananguchal (forests in river floodplains), 10% is reserve territory, and 30% is stubble. Mainly the land is lowland (floodplain).

The community is composed of the founding families belonging to the Uitoto ethnic group who migrated from La Chorrera around 1982 in search of well-being or, for the most part, under pressure from the guerrillas [7]; this migratory phenomenon has continued, accompanied in recent times by the arrival of some settlers [5, 12]. The community occupies approximately 289 ha in the Indigenous Ticuna-Huitoto Reservation from Km 6 to 11 (area of 5560 ha), which was constituted through Resolution 025-1978 of the *Instituto Colombiano de la Reforma Agraria* (Colombian Institute of Agrarian Reform INCORA) as a special reserve zone and, later, through Resolution 005-1986 INCORA, conferred upon it the legal status of a *Resguardo* [5, 7]. The area has small stores within the community or at the Km 6 community (1.5 km away) and markets in Leticia (7.9 km away).

### Sampling

Our ethnobotanical approach included sampling inside the Ziora-Amena community, the study zone was defined as a circle with a 10-km radius centered on the *Maloka Hitoma (Jitoma)*, since culturally, to establish a chagra, one looks for an area of forest or stubble (already abandoned) within the 2-km closest to the maloka or the house [7].

As inclusion criteria, we had people belonging to the community and who have a chagra; as exclusion criteria, we considered people who, at the time of the research, did not have their chagra active. The initial sampling yielded 23 families with chagra in the community (22 chagras had been reported in 2006 by Triana-Moreno and collaborators [5]); in applying the exclusion criteria, the sample was reduced to 14. For abundance assessment, an additional family was excluded as the visit to the chagra was not achieved after four attempts on different dates. The sample effectively represents a census of the active chagras in the community.

### Data collection

The research team conducted several visits to the community. Initially, the project was socialized and supported by the community in general and by the governors' council. Since this project developed a research issue that did not affect or interfere with the quality of life and practices of the community, the Colombian authority considered prior consultation not to be necessary (OFI18-14627-DCP-2500). Subsequently visits were made for the individual application of the data capture instrument.

The initial application of a preliminary instrument to the community in general (in the project’s socialization session), to some members of the *cabildo* and to some knowledgeable grandparents, allowed us to establish the inventory of existing chagras in the community. For each selected chagra, the person or persons directly responsible for its maintenance were interviewed. The participants were up to two persons in each chagra. The information on the use of the plants was collected with reference to their common name (vernacular or regional, e.g., *yucca, pineapple*), and the results are presented using these common names because these data do not always coincide with the individual botanical species one by one (in some cases, a folkloric taxon is used as the determinant of an entire botanical family) [10]. An ethnobotanical walk was carried out, tending to locate reported species; our team identified the majority of plants mentioned by the participants, by using pertinent floras, monographs [14], and the *Herbario Nacional Colombiano* (COL) [15]; specimens that were difficult to identify were sent to appropriate specialists for determination; vouchers were deposited in COL.

In order to minimize the possibility of one participant’s responses directly influencing those of another [16], in almost all cases (> 85%), participants were interviewed individually; additionally, and in order to avoid potential incentives to embellish the information or take on the interview as a task, data collection was made as informal as possible, fitting in when informants had free time and compensating participants for their time; at the end of the interview, with a tool for use in the chagra, the cost of which roughly equaled the monetary subsistence value of the time the participant gave to the project [16].

### Data analysis

#### Social and preference factors in the offer of non-timber forest products

The interest and expectations of the community in relation to the management of the resource, was studied from the context in which was formulated the question: "Which plants of the chagra, do you consider that can be more easily commercialized (trade in Leticia, tourists, nearby communities)?” (Plants were enunciated in order of importance or preference for commercialization, being the first one the most important or more recommended by the respondent). The place occupied by each taxon was converted into a score, and the Rapid Informant Rank (RIR) values were calculated [17] following the procedure established by Lawrence et al. [10] with some variations: for each species, we averaged the scores by sex; thus, for a given taxon (T), we specified its value attributed by females (f) or by males (m) as [RIR_Tf_ = ∑(*T*_f_/n_f_) or RIR_Tm_ = ∑(*T*_m_/n_m_)]. Finally, the RIR attributed by the whole sample in the community is calculated as [RIR_T_ = ½ (∑(*T*_f_/n_f_) + ∑(*T*_m_/n_m_))]. The applied instrument also explored the traditional knowledge associated with the time of seeding and harvesting, the need for co-seeding with another species, and the requirement of a specific place within the chagra, as well as whether there are cultural restrictions to the commercial exploitation of the species.

#### Use value

To categorize the uses reported by respondents, the following use categories were established: Food/Edible, Artisanal, Sawmill, Dye, Fuel, Construction, Cultural/Mythical, Forage, Medicinal, Ornamental, Psychotropic, Toxic/Poison, Sales/Trade, Other [6, 18, 19]. Each of the species, originally reported by the informant, was classified according to its use category, and based on this, the Use Value Index (UVI) was determined according to Hoffman and Gallaher [17]. Each taxon mention was registered separately and called as event, then the same species and the same participant can generate several events [16]; the number of events of a species (e) is added up within each use category and divided by the total number of events as follows: UVIe = (ΣUe)/(ne), where ne is the number of events in which the participant cites a use for the species (e). To ensure that the participant and the interviewer were talking about the same organism and to refresh the memory of the participant, a visit to the chagra was made during the interview.

#### Abundance

The presence and abundance of the reported plant species in the active chagras was evaluated following previously described procedures [6, 11], with some modifications; briefly, in the chagra, two fixed-width linear transects of 40 × 2 m (80 m^2^), arranged in the form of an “L” were carried out; in each one, information was collected on the population density of the 10 plant species previously reported by the participant; additionally, an ethnobotanical walk was carried out, tending to locate those species that, according to traditional knowledge, are located in particular areas of the chagra or in the stubble. The data obtained from the number of individuals observed in each transect of each chagra, were averaged, and the calculation per hectare was done [11]. Subsequently, the absolute frequency was calculated, understood as the degree of uniformity with which individuals of the same species are present in the evaluated chagras, using the formula: Fa = Pi / Np; where Pi corresponds to the number of plots in which the species (i) appears and Np corresponds to the total number of chagras inventoried, equally the relative frequency corresponds to Fr = (Fa / ∑Fa) × 100 [20].

### Economic and market differentiation factors

The business potential was assessed based on the knowledge and experience of a business stakeholder of the area who, based on the results of previous indicators of preference, abundance, and use, expressed a concept of prioritization of species. Also, as desirable characteristics of selection of prioritized plants, the equipment, together with the business stakeholder in the region, considered the perception of commercial factors (profitable marketing prices, affordable production costs, and ease of transport), social factors (traditional use in the chagra, absence of cultural restrictions for commercial exploitation), ecological factors (frequent harvesting, easy growth, possibility of multiple uses, sustainability in use, presence and abundance of the species in the active chagras, resistance to pests), and market differentiation factors (endemic species of the region and species without competition from large producers in the area) [11].

### Correlations among indicators

Data of index (RIR, UVI, and abundance) were submitted to regression analysis in order to evaluate the relationships in between. Also, prioritization index assigned by participants (RIR) face to prioritization assigned by the business stakeholder were submitted to regression analysis in order to assess relationships.

## Results

### Demographic characterization

**Fig. 2:**
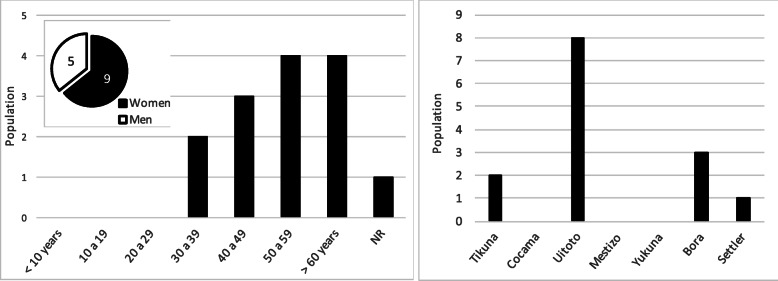
Characterization of the sample studied. *NR* no report

The selected sample was composed of 14 voluntary participants, corresponding to 13 different chagras, one of which could not be the object of the floristic observation; the sample corresponded to 93% of the active chagras existing at the time of the study (confidence level 95%, sampling error 6%). Seventy-one percent of the chagras visited have an active land less than 1 ha, and only four have an area greater than 1 ha (coincidental finding with the previous report by Triana-Moreno and collaborators [5]). Sixty-four percent of participants were female, 62% of the sample were 50 years old or older at the time of the interview, and no participants under 38 years of age were recorded. The Uitoto ethnic group predominates with 57% of the participants, followed by the Bora and Ticuna ethnic groups (Fig. 2).

Thirty-six percent of the participants surveyed are natives of the community or come from municipalities in the State of Amazonas (cities of Leticia or Puerto Nariño); the rest are foreigners to the state; 36% of them are natives of the region (from Putumayo State) and one respondent from the State of *Huila* (outside the region). Half of respondents had as their previous residence outside the State of Amazonas, mainly Puerto Leguízamo (State of Putumayo). The predominant marital status in the sample was voluntary union.

### Traditional knowledge (TK) transmission

Forty-three percent of participants acquired their TK on plants from maternal line (mother, grandmother, aunt), the rest acquired it from their father (29%) or both parents (21%) and one of them declared to be autodidact. All of the interviewees acquired their TK on the chagra, and 85% of them have (at the time of the interview) the chagra as their main job.

### Social and preference factors in the offer of agroforestry products

The sample of farmers reported experience in the cultivation of the referred species, for which, in general, there are no cultural limitations to commercial exploitation (99% of the 121 events registered); only in one event of four reported the *coca*; cultural restrictions were manifested for being a sacred plant. TK in the management of the chagra related to the need to crop it with another vegetable species (as a strategy of plant protection); the need to seed the vegetable species at a certain time, and the distribution of the vegetable species in the area of the chagra are typical of the management of the chagra and evident in the open conversations held during the visit to the chagras, more than in the structured instrument applied. It should be noted that this knowledge is maintained mainly by the older “grandparents”. Since the transmission of this knowledge is by oral tradition and the new generations do not show interest in it, an imminent risk of loss of this TK is consolidated.

A total of 38 different taxa were reported by 13 participants from 12 active chagras at the time of the study (Table 1).

**Table 1:** Species, reported by the participants of the chagras of the Ziora-Amena community Km. 7 of Leticia, Colombia

Names		Botanical family	Current distribution	RIR	UVI	Abundance
Common	Scientific					
Ají	*Capsicum spp* ^a^	Solanaceae	Neotropical	0.48	0.14	438
Algodón	*Gossypium barbadense* L.	Malvaceae	Cosmopolitan	0.39	0.07	250
Arazá	*Eugenia stipitata* McVaugh	Myrtaceae	Neotropical (Amazonian)	3.42	0.57	292
Asai	*Euterpe precatoria* Mart.	Arecaceae	Neotropical	1.69	0.29	792
Batata	*Ipomoea batatas* (L) Lam.	Convolvulaceae	Cosmopolitan	0.20	0.07	0
Bejuco (Sacha inchi)	*Plukenetia volubilis* L.	Euphorbiaceae	Neotropical (Amazonian)	1.33	0.14	375
Borojó	*Borojoa patinoi* Cuatrec.	Rubiaceae	Neotropical	2.68	0.50	700
Caimo	*Pouteria caimito* (Ruiz & Pav.) Radlk.	Sapotaceae	Neotropical	1.90	0.21	188
Camu camu	*Myrciaria dubia* (Kunth) McVaugh	Myrtaceae	Neotropical	0.56	0.07	500
Canangucho (aguaje)	*Mauritia flexuosa* L.f.	Arecaceae	Neotropical	1.72	0.36	375
Carambola	*Averrhoa carambola* L.	Oxalidáceae	Neotropical, Paleartic	0.33	0.07	375
Chontaduro	*Bactris gasipaes* Kunth	Arecaceae	Neotropical	3.69	0.71	786
Coca	*Erythroxylum coca* Lam.	Erythroxylaceae	Neotropical	0.78	0.43	2833
Coco	*Cocos nucifera* L.	Arecaceae	Cosmopolitan	0.70	0.07	250
Copoazú	*Theobroma grandiflorum* (Willd. ex Spreng.) K.Schum.	Malvaceae	Neotropical	4.03	0.57	1143
Daledale	*Calathea allouia* (Aubl.) Lindl*.*	Marantaceae	Neotropical	0.60	0.07	0
Dr+ma	*Ruellia aff. Malacosperma* Greenm.	Acanthaceae	Neotropical	0.28	0.07	0
Granadilla	*Passiflora sp.*	Passifloraceae	Neotropical	0.17	0.14	125
Guama	*Inga edulis* Mart.	Fabaceae	Neotropical	1.91	0.43	625
Jidoro	*Rudgea* sp.	Rubiaceae	Neotropical	0.11	0.07	250
Limón	*Citrus limon* (L.) Osbeck	Rutaceae	Cosmopolitan	0.68	0.29	417
Limoncillo (hierba luisa)	*Cymbopogon citratus* (DC.) Stapf	Poaceae	Cosmopolitan	2.07	0.36	542
Lulo	*Solanum sessiliflorum* Dunal	Solanaceae	Neotropical	0.40	0.07	250
Macambo	*Theobroma bicolor Humb &* Bonpl*.*	Malvaceae	Neotropical	0.50	0.14	750
Mejorana	*Lippia alba* (Mill.) N.E. Br. ex Britton & P. Wilson	Verbenaceae	Neotropical, Nearctic, Australian	0.06	0.07	0
Mucuracaá (mucura)	*Petiveria alliacea* L.	Phytolaccaceae	Neotropical, Nearctic	0.44	0.07	1250
Ñame	*Dioscorea trifida L.f.*	Dioscoreaceae	Neotropical	1.11	0.21	625
Pimentón	*Capsicum annuum* L.	Solanaceae	Cosmopolitan	0.36	0.14	688
Piña	*Ananas comosus* (L.) Merr.	Bromeliaceae	Cosmopolitan	5.51	0.64	4107
Plátano	*Musa paradisiaca* L.	Musaceae	Cosmopolitan	2.59	0.29	1250
Sacha Ajo	*Mansoa alliacea Lam*	Bignoniaceae	Neotropical	0.56	0.07	750
Santa María	*Lepianthes peltata* (L.) Raf. ex R.A. Howard	Piperaceae	Neotropical	0.22	0.07	1625
Stevia	*Stevia rebaudiana* (Bertoni) Bertoni	Asteraceae	Neotropical	0.11	0.07	0
Tomate	*Solanum lycopersicum* L.	Solanaceae	Neotropical, Nearctic	0.10	0.07	750
Ucuye (Huevo de toro)	*Macoubea witotorum* R.E. Schult.	Apocynaceae	Neotropical	0.50	0.14	125
Umari	*Poraqueiba sericea* Tul.	Icacinaceae	Neotropical	1.33	0.36	583
Uva caimarona	*Pourouma cecropiifolia* Mart.	Urticaceae	Neotropical	3.44	0.57	393
Yuca	*Manihot esculenta* Crantz	Euphorbiaceae	Cosmopolitan	4.58	0.57	5107

Common names were checked in the database: common names of plants from Colombia [21]. Additionally, scientific names were confirmed at Missouri Botanical Garden [22] and in ICN-UNC [15]. Distribution was verified at Missouri Botanical Garden [22] [3, 6, 12, 21, 23–26].

^a^ Species grown in the Colombian Amazon are *Capsicum chinense* Jacq. (Ají amarillo o lulito), *C. frutescens* L (Ají malagueta), *C. annuum*, *C. baccatum* y *C. pubescens* [23, 26].

The top 10 species for commercialization, according to the rapid informant ranking (RIR), were in increasing order: *guama, limoncillo, plátano, borojó, arazá, uva caimarona, chontaduro, copoazú, yuca*, and *piña*. RIR values attributed to each plant are presented in Table 1. Regarding the perception of to whom the chagra plants can be sold, 79% of participants consider the Leticia market and tourists as the main trade niches.

### Use value

**Fig. 3:**
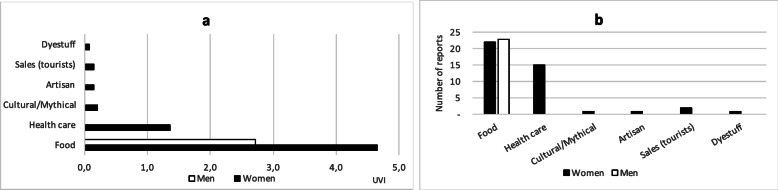
Use value index (UVI) and number of reports as a function of the major use class, in chagras of Ziora-Amena community. **a** UVI distribution, mentioned by women and men, according to general category of use. **b** Number of reports according to general category of use

Quantification of the multidimensional concept of cultural relative importance is a challenging task, in order to contribute to an approach as objectively as possible, in addition to the RIR, the use value index (UVI), by reported species, was assessed; Table 1 shows the UVIs of reported species. The distribution of UVIs, allocated by women and men to each general category of use, as well as the distribution of the number of reports in each category are shown in Fig. 3. On average, women reported nine different taxa each, while men reported eight.

### Abundance

**Fig. 4:**
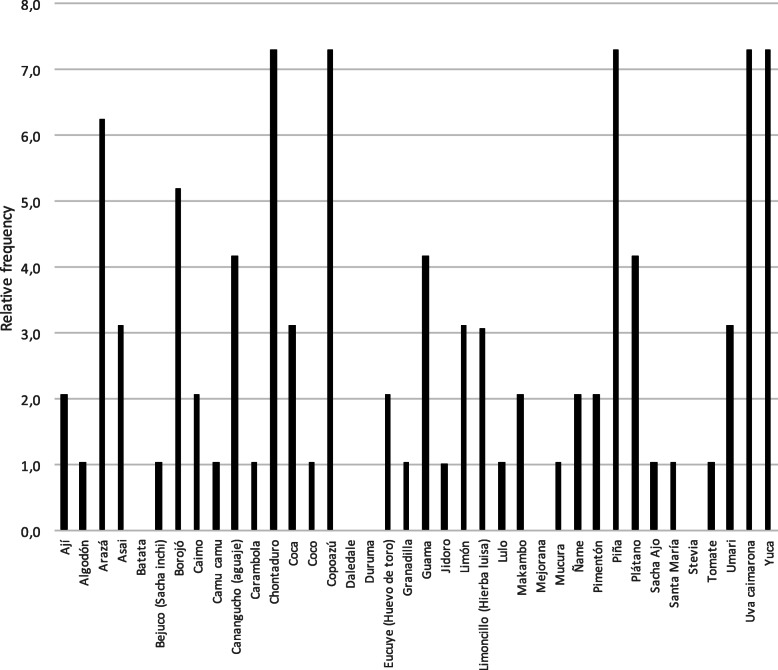
Relative frequency and uniformity of species appearance in the chagras of the Ziora-Amena community

Species found most abundantly in the conducted inventory are, in ascending order: *copoazú, mucura, plátano, santa maría, coca, piña*, and *yuca*, with over 1000 specimens per hectare. The abundance of the reported species is presented in Table 1. The relative frequency allows evidence in a greater number of chagras the species *chontaduro, copoazú, piña, uva caimarona*, and *yuca*, followed by *Arazá* and *borojó*; the remaining assessed species exhibit presence in less than 5 chagras, Fig. 4.

### Correlations among indicators

**Fig. 5:**
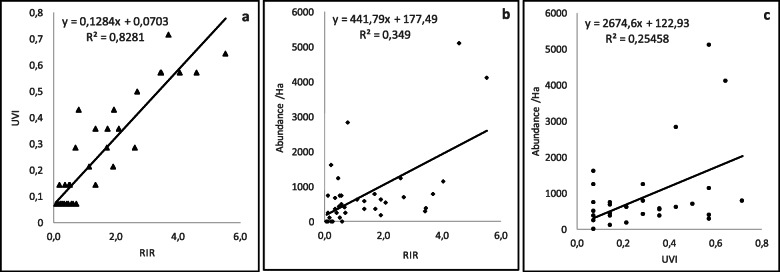
Correlation among perception, use, and abundance indicators

Regression analysis showed that there is a very good correlation (*R*^2^ = 0.83) between the species nominated as most important (RIR), and the value of use that the community gives to them (UVI) (Fig. 5a). However, this correlation is not so linear when the indicators of perception and use are evaluated versus the abundance found in the chagras (Fig. 5b and c).

### Economic and market differentiation factors

Based on indicators of preference (RIR), use value (UVI), and abundance, the team explored the perception of a business stakeholder in the area (with a background in managing an ecological park that constantly receives tourists with interest in products from the region). Technical feasibility and market differentiation factors were also considered. This led to the prioritization of species: *bejuco (sacha inchi), macambo, copoazú, mucuracaá (mucura), sacha ajo, uva caimarona, canangucho (aguaje), umari, guama*, and *jidoro*.

**Fig. 6:**
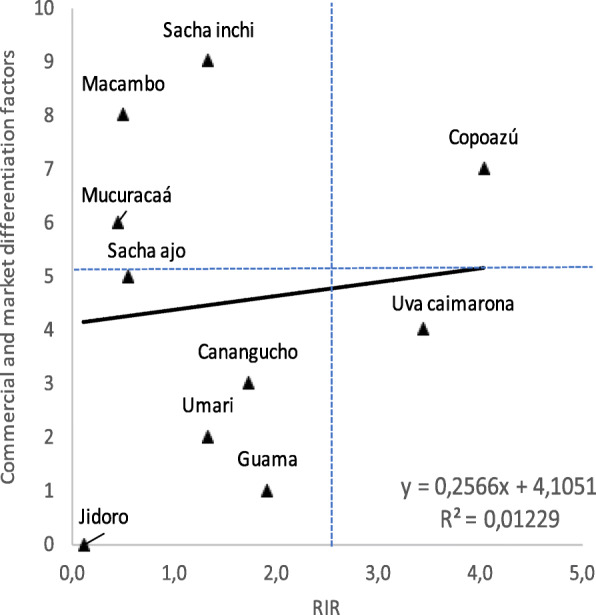
Correlation among perception indicators and commercial & market differentiation factors

General correlation between the perception indicator (RIR) and the commercial valuation and market differentiation made with a trading user of these goods is low (R^2^=0.01), however, there are coincidences in these two indicators (quadrants 2 and 3 Fig. 6) for the species *copoazú, sacha ajo, canangucho, umari, guama* and *jidoro*.

Our study prioritizes the plants: *bejuco (sacha inchi),* present in two of the chagras as a grass that grows robustly and abundantly; *macambo and copoazú,* two theobromas of important UVI in the community; *mucuracaá (mucura), sacha ajo, uva caimarona, canangucho (aguaje), umari, and guama* (native species of the Amazon); and *jidoro*, a plant reported by community as traditionally used in dyes for non-permanent tattoos.

A systematic review of the existing evidence of use, was developed for the prioritized species, and from this, a monographic chart was made for each one. The monographs were compiled in the brochure "Plants of interest of the chagra of the Ziora-Amena indigenous community" which will be returned to the community as one of the benefits of the research.

## Discussion

**Fig. 7:**
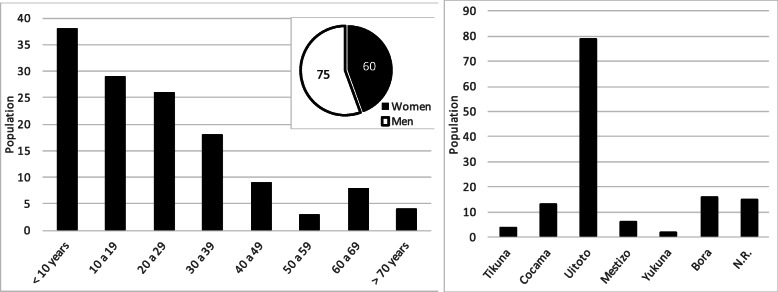
Characterization of the Ziora-Amena community [12]. *NR* no report

In 2008, the community had a population of 135 individuals who comprised approximately 37 families, where 44% were women; 50% of the population was under 19 years old, and only 9% were over 60; there were no people over 75 years old, and 59% of the population were Uitoto, (Fig. 7) [5, 12]. In the community, there is a perception of marked population growth in recent years, derived from migration from distant municipalities in search of opportunities in the city of Leticia; population projections developed by the National Administrative Department of Statistics of Colombia [27] estimate that the population of the Leticia municipality grew in the decade 2008 to 2018 by 8.5% from 38,957 to 42,280 inhabitants; the extrapolation of this growth to the community of Km 7 of the Leticia municipality indicates an estimated population of 147 people at the time of development of this study.

In general, the global characterization of community is reflected in the sample studied (Figs. 2 and 7); except in the age composition where the sample of active chagras owners, as opposed to the distribution of the general population (Figs. 2 and 7), shows an inversion of the graph, with adults over 50 years old (62% of the sample) predominating in the chagras, while in the general population, young people predominate. This situation may be due to the lack of interest of current generations in maintaining the traditional culture of the chagra, leaving it in the hands of the elderly, which allows us to see the risk of losing this traditional and sustainable practice. Finally, the low proportion of the population that is still dedicated to this traditional practice is evident (11 of approximately 37 families), possibly due to the proximity of this community to the city of Leticia where they work in various other jobs.

Mainly, the chagra of this community gives rise to a traditional domestic harvesting of non-timber forest products [20]; however, the needs of the population, related to acquisition of products from the family basket, not produced or insufficient in the area, have led to the development of a persistent use; since the subsistence of the inhabitants of the community depends mainly on fishing and the cultivation of the land, whose products also serve as merchandise for exchange in the local trade and in Leticia; with the money obtained, they complement the diet with the achievement of other products.

Traditional knowledge has been transmitted in indigenous peoples, from generation to generation, for thousands of years [28], in general, from parents to children, a situation that in this case was reported, highlighting additionally a high percentage (43%) of cases in which transmission occurs through maternal line (as described by other authors) since the chagra is considered a space of female domain fertility, complementary to maloka [4]. In this community, it was evidenced that elders (traditional grandparents) maintain the knowledge about the management of chagra and medicinal plants (finding consistent with previous reports) [3]; taxa registered in this last category were provided by this stratum of the sample; the other age strata exercise the handling of chagra through practices mixed with contributions from other cultural origins.

The seeding of a chagra is a cultural activity preceded by a spiritual preparation aimed at maintaining the teachings of traditional culture; it is also an activity of a ritual nature that is done through a *minga* (associated work journey) [3, 5]. Specific aspects of the organization and traditional management of the chagra, such as the sowing of crops, planting at specific times and the distribution of plant species in the chagra area, were more evident in the open interviews, in situ, with the participants than in the specific questions of the instrument applied, making it clear that this knowledge is intrinsic to the work of the chagra and that it is evident in the accompaniment of the different activities of the chagra.

In the 38 taxa reported by the participants, a predominance of vegetal species of neotropical distribution (66%) is evident; additionally, 10% are distributed in one or two biogeographical regions (additional to the neotropical one), and 24% of the taxa are distributed in more than four biogeographical regions, considering them cosmopolitan (Table 1); this predominance of species endemic to the neotropics, and some of them with a very reduced distribution in the Amazon, represents an opportunity as it is a market differentiation criterion that highlights the supply of goods with very particular characteristics that may be of great interest.

The nominated species belong to 28 different families within which the Arecaceae (palms) and Solanaceae stand out with a slight preponderance of four species reported in each, Euphorbiaceae, Malvaceae, Myrtaceae, and Rubiaceae, with two nominated taxa in each of them, and the remaining families are represented by only one species. The value of palms is outstanding since 3 of the 15 most valued taxa (RIR) are palms, being the most nominated family in this group (finding consistent with other authors) [10]. These findings make evident the biological diversity of the area and particularly of the chagras, which is a cornerstone of its strength, ensuring an adequate and balanced human diet and a lower risk of pests since the presence of different species makes it more difficult for insects and monospecific pests to dominate and spread explosively [3].

Through quantitative methods, we seek to obtain evidence about the relative values of the different plants by quantifying, in a relatively quick but socially sensitive way, the perceptions of the different stakeholders and then explore the different categories of use and their real abundance in the active farms at the time of the study. Participants’ perceptions of which chagra plants can be most readily traded are presented in Table 1 (RIR); these values are averaged over the entire sample (not simply over the participants who nominated each taxon), then influenced only by the frequency of nomination as each non-nomination contributes a score of zero to the overall average value. There is no difference in the RIR value between women and men (U Mann–Whitney test α = 0.05). In terms of the number of taxa, women nominated 84% of the 38 taxa reported, while men nominated only 61% (23 of the 38 taxa), derived from the fact that females represent 64% of participants interviewed and that, as previously mentioned, the chagra is considered a fertility space of female dominance. The species nominated only by women were *umarí, coca, camu camu, sacha ajo, makambo, eucuye, mucura, algodón, carambola, dr+ma, santa maría, granadilla, stevia, jidoro*, and *mejorana*, while those nominated exclusively by men were *caimo, coco, daledale, lulo, batata*, and *tomate*.

The use value index allows, by consensus of the participants, a quantitative approximation to the cultural relative importance (CRI) given to the nominated taxa, understood as a non-monetary concept. Structured instruments are more susceptible to statistical analysis than open-ended approaches; however, the use of survey type information collection instruments can limit responses to plant uses. In our case, participants provided information limited to taxa previously defined as most suitable for trade and assigned them to previously defined categories of use; these techniques are applied when time in the field is limited [17]. Grouping the specific uses cited by participants into use categories facilitates efficient compilation, comparison, and presentation of the data sets; however, it should be noted that plants that are frequently cited for similar uses (e.g., whole fruit for food or fruit for juice) may receive atypically high CRI scores, and the method does not distinguish between degrees of importance and analyzes only the average number of uses cited, so a rarely used plant with two cited uses would be better scored than a very popular plant with only one use [17, 19, 29]. The above approach is valid when studying useful plants in general; however, under this approach, the relative importance of chagra as a source of medicinal plants is usually underestimated, because medicinal plants obtain the lowest UVIs, and their scientific value can be lost [30]. The UVI score of selected taxa is presented in Table 1.

The prioritization of our study highlighted the *bejuco (sacha inchi)* that, in its seeds, has a high content of unsaturated fatty acids, particularly α-linolenic acid (omega-3) and to a lesser extent linoleic acid (omega-6) and omega-9 [31], whose properties in the reduction of risk of dyslipidemias by means of reduction of total cholesterol and LDL cholesterol in plasma, with increase of HDL cholesterol and proteins, have been described [32]. Species allows in an initial stage (early victory) to stimulate the production and sale of roasted seeds to tourists, as an alternative to recover the interest of the community in the chagra and thus preserve the associated TK. In later stages of development, this species allows for the production of *sacha inchi* oil, an alternative to fish oil that does not have the taste, considered unpleasant (and sufficient to reject its use) of the latter.

Next in the order of prioritization are the species *macambo* and *copoazú*, two theobromas that may give rise to exotic soft drinks to offer to tourists [33] and, in later developments, can give rise to raw materials of important added value, for the food and pharmaceutical industries [34–36]. *Mucuracaá (mucura)* is a plant of therapeutic use accepted as an anti-inflammatory of the oral mucosa, coadjutant in the treatment of inflammatory processes [37, 38]. *Sacha ajo, uva caimarona, canangucho (aguaje), umari*, and *guama* are exotic species from the Amazon offering opportunities both for sale on the local market and to tourists. Finally, *jidoro*, used in dyes for non-permanent tattoos, which can be the basis of a tattoo offer to tourists and has great potential as a colorant for food and pharmaceutical industries.

## Conclusions

The chagra of Ziora-Amena community of Km 7, as a traditional agroecosystem adequate to the Amazonian environment, is in danger due to the centralization of its handling only in the stratum of older adults of the community, the transmission by oral tradition of its traditional knowledge (and the disinterest of younger generations in the same), and the competition of the market and labor scenario offered by the nearby city of Leticia.

The chagras of community have great biodiversity, the perception of participants allowed to highlight 38 taxa of interest for their commercialization, which belong to 28 different botanical families, predominating the neotropical species.

Regarding the perception of marketing scenarios for the chagra plants, 79% of the participants consider the Leticia market and sales to tourists as their main marketing opportunities. Indicators of perception, use and abundance show preference and predominant use of food species.

Alternatives are needed that, in keeping with the social context and opportunities of the area, allow the recovery of interest in the chagra and thus preserve the traditional knowledge at risk today. Research on plant species endemic to the region, with property of reducing health risks, or ingredients for the food or pharmaceutical industry, represent an opportunity of high added value for the region.

## Data Availability

Further information is available from the corresponding author on reasonable request.
